# The Effect of N-acetylation and N-methylation of Lysine Residue of Tat Peptide on its Interaction with HIV-1 TAR RNA

**DOI:** 10.1371/journal.pone.0077595

**Published:** 2013-10-17

**Authors:** Santosh Kumar, Souvik Maiti

**Affiliations:** 1 Proteomics and Structural Biology Unit, CSIR-Institute of Genomics and Integrative Biology, CSIR, Delhi, India; 2 CSIR-National Chemical Laboratory, Dr. Homi Bhabha Road, CSIR, Pune, India; George Mason University, United States of America

## Abstract

Post-translational modification (PTM) of RNA binding proteins (RBPs) play a very important role in determining their binding to cognate RNAs and therefore regulate the downstream effects. Lysine can undergo various PTMs and thereby contribute to the regulation of different cellular processes. It can be reversibly acetylated and methylated using a pool of respective enzymes, to act as a switch for controlling the binding efficiency of RBPs. Here we have delineated the thermodynamic and kinetic effects of N-acetylation and N-monomethylation of lysine on interaction between HIV-1 TAR RNA and its cognate binder Tat peptide ( a model system). Our results indicate that acetylation of lysine 50 (K50), leads to eight- fold reduction in binding affinity, originating exclusively from entropy changes whereas, lysine 51 (K51) acetylation resulted only in three fold decrease with large enthalpy-entropy compensation. The measurement of kinetic parameters indicated major change (4.5 fold) in dissociation rate in case of K50 acetylation however, K51 acetylation showed similar effect on both association and dissociation rates. In contrast, lysine methylation did not affect the binding affinity of Tat peptide to TAR RNA at K50, nonetheless three fold enhancement in binding affinity was observed at K51 position. In spite of large enthalpy-entropy compensation, lysine methylation seems to have more pronounced position specific effect on the kinetic parameters. In case of K50 methylation, simultaneous increase was observed in the rate of association and dissociation leaving binding affinity unaffected. The increased binding affinity for methylated Tat at K51 stems from faster association rate with slightly slower dissociation rate.

## Introduction

Post-translational modifications (PTMs) of RNA binding proteins (RBPs) regulate many important cellular functions including nuclear transport, various signaling pathways and gene expression during stress conditions [1-3]. Most of the RBPs are rich in positively charged amino acids which mediate the initial recognition of the negatively charged phosphate backbone of the RNA followed by the formation of specific H-bonds mediated by side chain, terminal protons of amino and guanidinium groups respectively for lysine and arginine [4,5]. Any alteration in this molecular arrangement would result in a change in binding strength of RNA and protein. PTM of lysine and arginine perform regulatory functions at both, DNA as well as RNA level through transcriptional alterations. Lysine methylation and acetylation, prominent in histone protein [6,7], are the most common and extensively studied PTM that regulates the cellular processes. Here lysine acts as a master controller, and undergoes various PTMs to modulate the expression levels of the related genes [8-10]. Histone methyl transferases and histone acetyl transferases are responsible for the methylation and acetylation of the lysine residues of histone proteins respectively [11,12]. These methyl transferases can produce mono, di or tri-methylated lysine depending on the specific methyl transferases involved [13]. Lysine methylation has been linked to a plethora of disease conditions related to growth and development of the organism, where levels of these enzymes are altered [14].

 Human immunodeficiency virus (HIV-1) contains many regulatory proteins which undergo PTMs to modulate the rate of transcription, mRNA transport and other processes. One of the key regulatory proteins Rev mediates the export of unspliced mRNA from nucleus to the cytoplasm [15,16]. Methylation of Rev protein at arginine residue inhibits its binding to Rev response element (RRE) of RNA and thereby hampers its export from nucleus to cytoplasm [17]. Integrase, an enzyme, that catalyzes the integration of viral DNA (produced after reverse transcription) to the host DNA, also undergoes lysine acetylation and enhances the 3´-end processing and strand transfer reaction [18]. Notably another important regulatory protein Tat, known to regulate the rate of transcription elongation also undergoes extensive PTMs [19-21].

Tat protein of HIV-1 aids propagation of virus in the host cell. It is necessary for the assembly of various transcription co-activators such as positive transcription elongation factor b (PTEFb), which contains CDK9/CyclinT1 and histone acetyl transferases [22]. Tat has also been shown to regulate the activation of C-terminal domain kinase in phosphorylation of C-terminal domain of RNA pol II [23]. Most importantly, arginine rich motif of Tat protein interacts with the stem loop structure of transactivation region (TAR) of mRNA and regulates the rate of transcription elongation. 

Arginine rich motif of Tat contains the positively charged amino acids which mediate its binding to TAR RNA. Mostly arginine and lysine amino acids are involved in direct hydrogen bond formation to the RNA, and modification at these amino acids interfere with their configuration and thereby alters the interaction of Tat protein with TAR RNA. Arginine can undergo methylation at different nitrogen atoms of guanidinium group, depending on the enzymes involved [24]. Protein arginine methyl transferase 6 (PRMT6) is known to catalyze the asymmetric dimethylation of Tat protein at arginine, which negatively affects its interaction with TAR RNA and cyclinT1 protein [25]. Arginine methylation in Tat protein is also known to increase the *in vivo* stability of the protein [26]. Apart from arginine Tat protein also contains many lysine residues which are critical for transactivation of TAR RNA. These lysines are mainly present in the basic binding domain of Tat protein i.e. 49-57 residue, and are known to be methylated as well as acetylated at K50 and K51 positions [27-30]. In vitro and cell culture based studies have demonstrated that acetylation of K50 and K51 of Tat hampers the production of full length infectious virions [31]. On the contrary monomethylation at K51 by lysine methyltransferase Set7/9-KMT7 results in enhancement of HIV transcription [27].. Both acetylation and methylation act as a switch to regulate the gene expression either positively or negatively depending on the type of modification [32]. We have chosen Tat as our model peptide to study the effect of lysine acetylation and methylation on RNA binding because it is biochemically and structurally well-studied system [33-35]. 

 Herein we have modified (N-acetylation and N-methylation) lysine residues of Tat peptide at different positions (K50 and K51) and performed the biophysical experiments to calculate the effect of these modifications on the binding properties and thermodynamics of interaction between Tat peptide and HIV-1 TAR RNA. Melting experiments were performed to study the effect of the modifications on the thermal stability of the RNA peptide complex. Variations in structure, thermodynamic and kinetic parameters were further measured using circular dichroism (CD), isothermal titration calorimetry (ITC) and surface plasmon resonance (SPR) experiments, respectively.

## Material and Methods

### Materials

**Figure 1:**
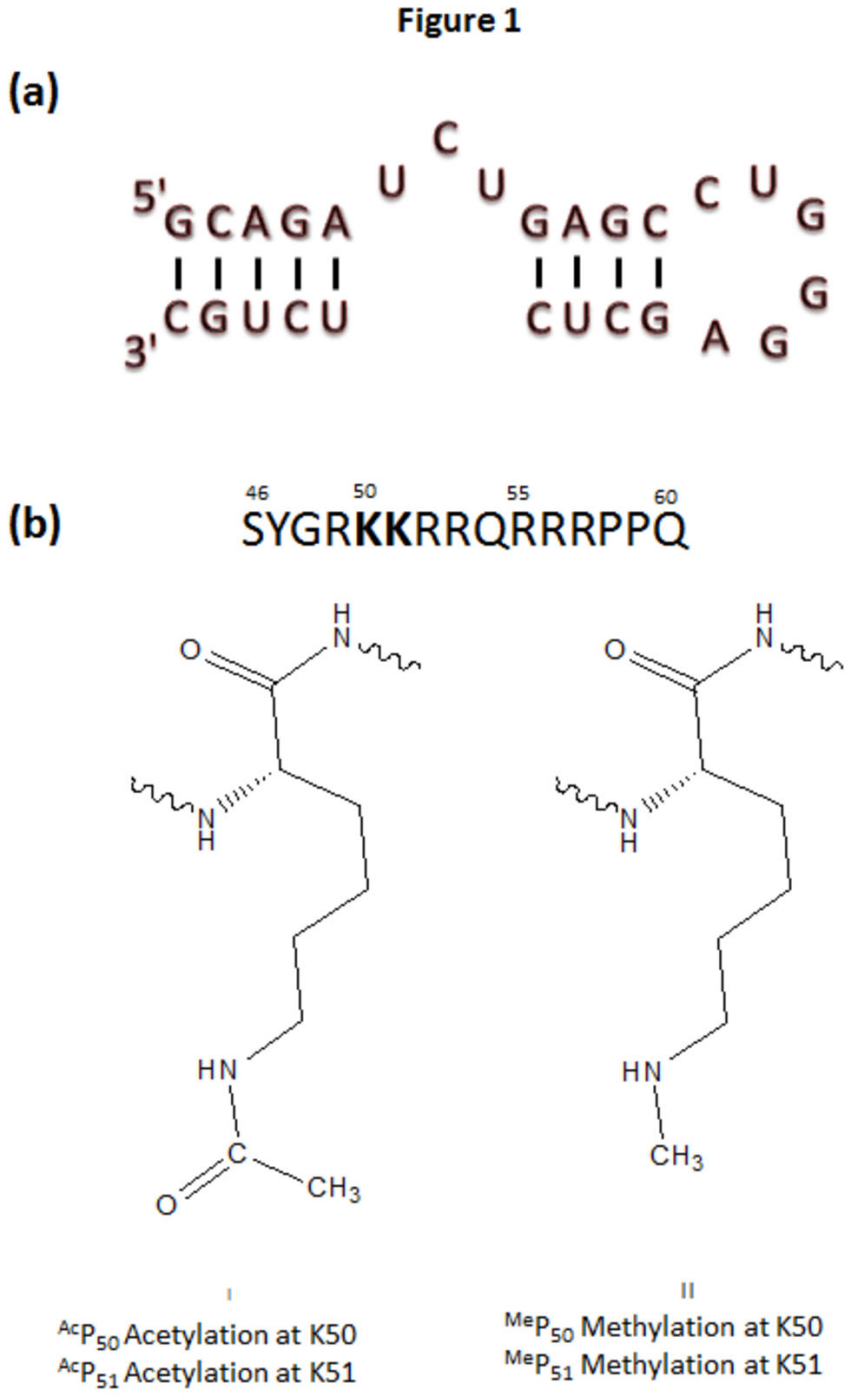
The sequence of (a)TAR RNA and (b) Tat peptide along with different lysine modifications and their positions.

The sequence of HIV-1 Tat peptide (SYGRKKRRQRRRPPQ), along with different modified peptides (shown in Figure 1b) used for these studies, were purchased from GL Biochem. (Shanghai) Ltd. and used without any further purification. HPLC purified 27 nucleotide long stem loop (shown in Figure 1a) of HIV-1 TAR RNA (5´-GCAGAUCUGAGCCUGGGAGCUCUCUGC-3´) was obtained from Sigma Aldrich. The concentration of oligonucleotide was determined spectrophotometrically at 260 nm using the molar extinction co-efficient 251800 M^-1^cm^-1^. These values were calculated by extrapolation of the tabulated values of dimer and monomer nucleotides at 25 °C to high temperatures using previously reported protocol [36]. 

All stock solutions were prepared in the Milli-Q water. Other reagents of analytical grade quality were used without any further purification. All experiments were performed using 10 mM sodium cacodylate buffer containing 0.1 mM EDTA and 70 mM NaCl at pH 7.5.

### CD Spectroscopy

Circular Dichroism (CD) spectra were recorded on a Jasco spectropolarimeter (model J815, Japan) equipped with a thermoelectrically controlled cell holder. The cuvette used for acquiring spectra was of 1 cm in path length. CD spectra were recorded from 320 nm to 200 nm, with averaging of 3 accumulations at 25 °C. Each spectrum was collected at a rate of 100 nm/min, and 1 nm data pitch. The buffer solution contained 10 mM sodium cacodylate buffer, 0.1 mM EDTA and 70 mM NaCl at pH 7.5. For all the CD experiment RNA concentration was fixed at 2 μM and peptides concentration were 10 μM.

### Temperature dependent UV Spectroscopy (UV melting)

 Temperature dependent UV melting experiments were carried out using a Cary 100 (Varian) Spectrophotometer equipped with thermoelectrically controlled cell holder. A quartz cell of 1 cm path length was used for all absorbance studies. Temperature dependent absorption spectra were obtained at 260 nm with 0.5 °C/min. rate of increase in temperature, from 30 to 95 °C. In these experiments the concentration of RNA was kept 1 μM and peptide concentration was increased from 0-3 μM. The buffer solution contained 10 mM sodium cacodylate buffer, 0.1 mM EDTA and 70 mM NaCl at pH 7.5. For each optically detected transition, the melting temperature (*T*_*m*_) was determined using previously described methods [37].

### Isothermal Titration Calorimetry (ITC)

 Isothermal titration calorimetry measurements were conducted at 25 °C on a Microcal VP-ITC (Microcal, Inc.; Northampton MA). Titration of HIV-1 TAR with Tat and different modified peptides were done by injecting 4 μL aliquots of 350 μM peptide from a 250 μL rotating syringe (350 rpm) into an isothermal sample chamber containing 1.5 mL HIV-1 TAR RNA solution that was 10 μM in strand. Each experiment of this type was accompanied by the corresponding control experiment in which 350 μM peptide was injected into a solution of buffer alone. The duration of each injection was 8 s and the delay between the injections was 180 s, the initial delay prior to the first injection was 300 s. First injection of each titration was used as dummy injection and was removed from the analysis. Each injection generated a heat burst curve (microcalories/second vs. seconds) and the area under each curve was determined by integration [using the origin version 7.0 software (Microcal, Inc.; Northampton, MA)] to obtain the measure of the heat associated with that injection. The buffer corrected ITC profiles for the binding of Tat and different modified peptides were fit with a model for two set of binding site [38]. The net enthalpy change for HIV-1 TAR RNA stem loop interaction with Tat and different modified peptides were determined by subtraction of the heat of dilution. The binding parameters obtained from these fits are listed in the Table 1. The differential binding parameters for modified peptides with respect to wild type Tat peptide were calculated and are listed in Table 2. 

**Table 1:** Thermodynamic parameters of Tat peptide and modified peptides binding to HIV-1 TAR RNA obtained from ITC experiments at 25 °C.

**Peptide**	***n*_1_**	**^a^Δ*T*_*m*_ (°C)**	***K*_*A1*_ × 10^6^ (M^-1^)**	**Δ*H*_*1*_ kcal/mol**	***T*Δ*S*_*1*_ kcal/mol**	***n_2_***	***K*_*A2*_ × 10^4^ (M^-1^)**	**Δ*H*_*2*_ kcal/mol**	***T*Δ*S*_*2*_ kcal/mol**
^b^Tat	0.9	3.0	19.0 ± 0.1	-5.1 ± 0.1	4.7 ± 0.1	1.6	50.0 ± 0.4	-2.0 ± 0.2	5.7 ± 0.1
^Ac^P_50_	1.0	2.3	2.3 ± 0.5	-5.2 ± 0.2	3.4 ± 0.2	1.4	8.8 ± 0.5	-4.8 ± 0.4	1.8 ± 0.1
^Ac^P_51_	1.0	2.7	5.6 ± 0.4	-8.8 ± 0.3	0.3 ± 0.1	1.2	6.4 ± 0.6	-5.4 ± 0.1	1.1 ± 0.2
^Me^P_50_	1.1	3.1	15.0 ± 0.9	-8.8 ± 0.2	0.9 ± 0.1	2.4	12.0 ± 0.3	-3.2 ± 0.4	3.6 ± 0.3
^Me^P_51_	1.0	4.0	59.0 ± 0.9	-8.1 ± 0.1	2.4 ± 0.1	2.8	34.0 ± 0.5	-3.0 ± 0.1	4.4 ± 0.2

All the binding isotherms were analyzed using two set of binding sites model. ^a^Melting temperature, *T*_*m*_ were calculated from UV-melting experiments using method described by Marky *et al.* [37].

**Table 2:** Differential thermodynamic data of various modified peptide relative to the wild type Tat peptide.

**Peptide**	***KA1*/*K*_*A1*_^Tat^**	**ΔΔ*H*_*1*_ kcal/mol**	***T*ΔΔ*S*_*1*_ kcal/mol**	***KA2*/ *K*_*A2*_^Tat^**	**ΔΔ*H*_*2*_ kcal/mol**	***T*ΔΔ*S*_*2*_ kcal/mol**	**ΔΔ*T*_*m*_ (°C)**
Tat	1.00	0.0	0.0	1.00	0.0	0.0	0.0
^Ac^P_50_	0.12	-0.1	-1.3	0.16	-2.8	-3.9	-0.7
^Ac^P_51_	0.29	-3.7	-4.4	0.12	-3.4	-4.6	-0.3
^Me^P_50_	0.78	-4.0	-0.9	0.24	-1.2	-2.1	0.1
^Me^P_51_	3.10	-3.0	-2.3	0.68	-1.0	-1.3	1.0

All the diiferential binding paprameters were calculated using following equations. ΔΔ*H* = (Δ*H* ^p^ – Δ*H* ^Tat^), *T*ΔΔ*S* = (*T*Δ*S* ^p^ - *T*Δ*S* ^Tat^), ΔΔ*T*_*m*_ = Δ*T*_*m*_^p^ - Δ*T*_*m*_^Tat^) respectively.

### Surface plasmon resonance (SPR)

All kinetic parameters for binding of the peptides to RNA were calculated from SPR experiments performed on BIAcore 3000 system running with BIAcore 3000 control software version 4.1.2. The TAR RNA was biotinylated at the 5´-end with a 10 mer poly T linker attached to the 5´-end of the RNA. The 100 nM stock solution of RNA was used for immobilization on the flow cell 2 of streptavidin coated SA chip until an RU change of 500-600 was achieved. After immobilization the buffer was allowed to flow on the sensor chip surface to remove any unbound RNA. Flow cell 1 was left blank to account for nonspecific background signal, and was subtracted from the signal in flow cell 2. All the RNA and peptide stock solutions were prepared in 10 mM sodium caodylate buffer with 0.1 mM EDTA and 70 mM NaCl in the presence of 0.005% Igepal at pH 7.5 and serial dilutions of the 10 μM stock were done to make their concentration series. The binding and dissociation each was monitored for 300 s, followed by 60 s regeneration using buffer, which contained 1M NaCl and 50 mM NaOH. The sensograms were analysed with BIAevaluation software version 4.1.1 using two independent binding site model and the goodness of the fitting was monitored by χ^2^ value. 

## Result

### CD Results

**Figure 2:**
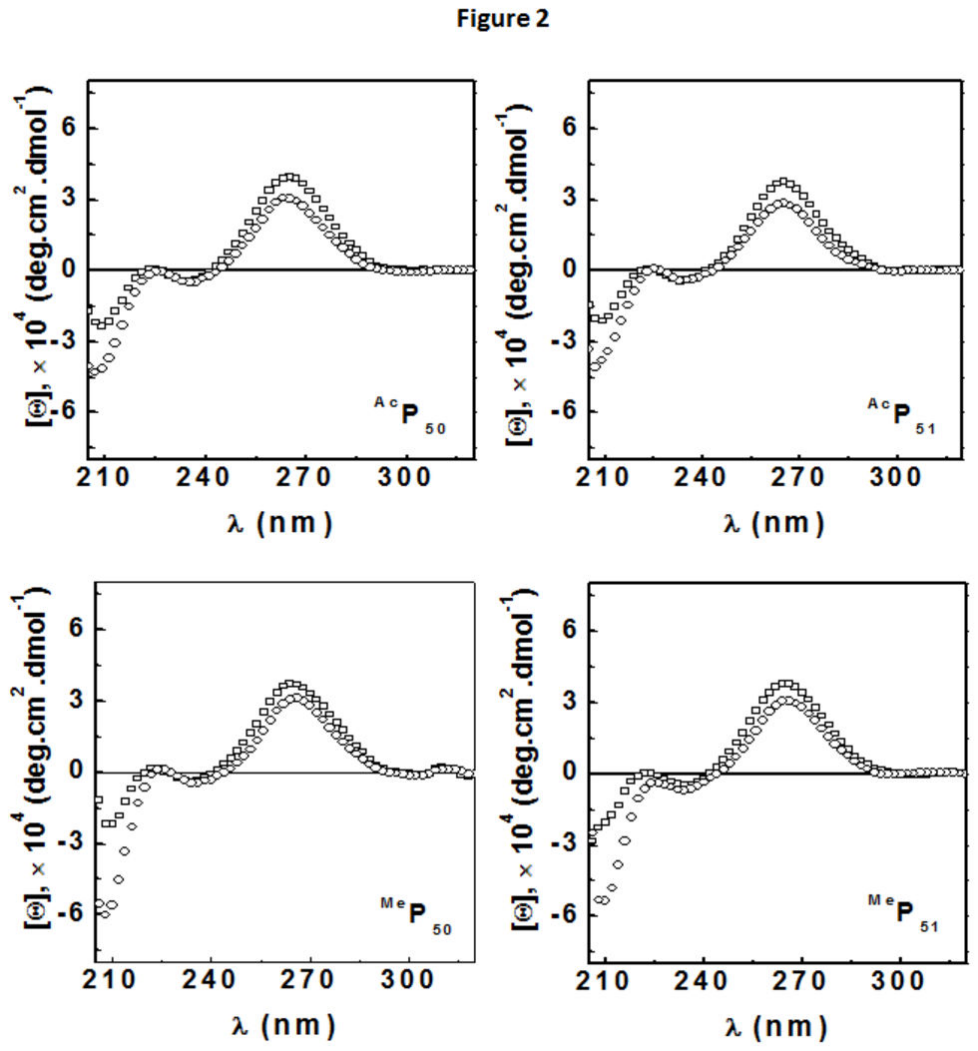
CD spectra TAR RNA and the effect of binding of different peptides on the structure of the RNA at 25 °C. (□) 2 μM RNA, (○) 10 μM Peptide. All CD spectra were collected in a buffer containing 10 mM sodium cacodylate, 70 mM NaCl and 0.l mM EDTA at pH 7.5.

CD spectra of TAR RNA were collected in the absence and presence of different modified peptides. The CD spectrum of RNA in the absence of peptide showed a positive maxima at 267 nm, and negative minima at 235 nm as well as 210 nm, Figure 2 (□). The minima at 210 nm was more negative compared to 235 nm minima. CD signal at these wavelengths represent characteristic signature of A-form of RNA. Addition of 5:1 molar ratio of peptide to RNA (all cases of modified peptides) resulted in decrease in amplitude of 267 nm signal indicating small destacking while the signals at 235 nm and 210 nm became more negative (Figure 2 (○). All the four modified peptide binding showed structural changes in the TAR RNA similar to the changes brought upon binding of wild type Tat peptide [39]. Thus these results indicate that lysine acetylation and methylation at different positions (K50/K51) exhibit structural changes similar to that of the wild type Tat peptide and no differential change was observed among them. 

### UV melting

The arginine rich motif of Tat protein (Figure 1b) contains mostly positively charged amino acids that play a crucial role in determining the binding of the protein to its cognate RNA partner. We have selectively chosen lysine residues of the ARM, known to be important for RNA binding [40], and modified them sequentially. Different modifications and their positions have been summarized in Figure 1b. Temperature induced melting experiments were performed to see the effect of various lysine modifications on the stability of RNA-peptide complex. Increase in temperature, by facilitating the opening of RNA duplex, leads to hyperchromicity. The unfolded fraction (α_unfolded_) can be calculated from the hyperchromicity change and plotted against temperature to calculate melting temperature (*T*_*m*_) of the RNA [37]. 

**Figure 3:**
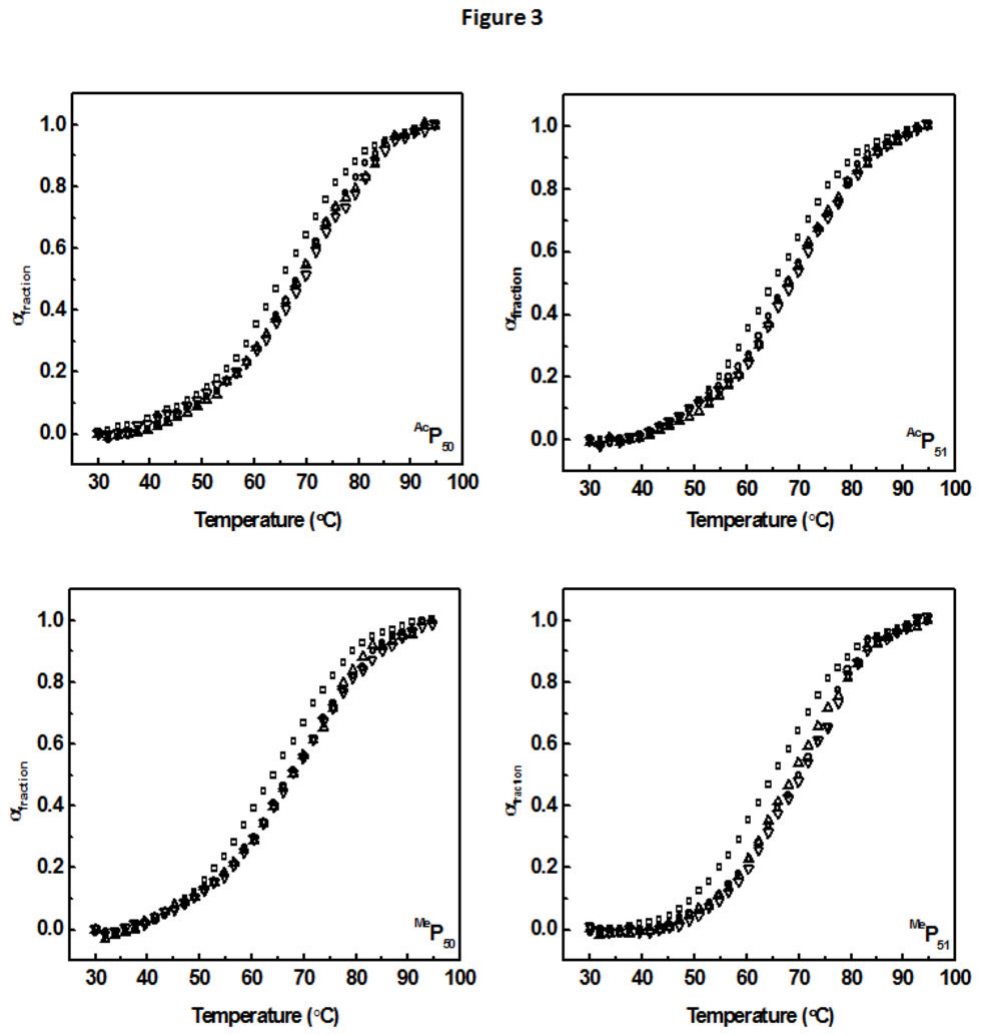
Thermal melting profile of TAR RNA alone and in the presence of different molar ratio of the peptides. (□) 1:0 molar ratio RNA:peptide (○)1:1 molar ratio RNA:peptide (△)1:2 molar ratio RNA:peptide (▽)1:3 molar ratio RNA:peptide. All the melting profiles were collected at 260 nm wavelength. The buffer condition were same as described in Figure 2 caption.

The *T*_*m*_ of HIV-1 TAR RNA in absence of peptides was 66.7 °C, and found very similar to the previously reported value [39]. Tat peptide binding enhances the stability of the TAR RNA secondary structure by increasing the *T*_*m*_ to 69.7 °C [39]. Various modifications of lysine have differential effect on the RNA structure stability. ^Ac^P_50_ peptide stabilizes the complex by 2.3 °C (Figure 3, ^Ac^P_50_), at 1:1 peptide to RNA molar ratio whereas at higher molar ratios, it showed marginal stabilization similar to the wild type Tat peptide binding. Similarly ^Ac^P_51_ exhibits 2.7 °C stabilization (Figure 3, ^Ac^P_51_). This indicates, acetylation of lysine increases the stability of the complex in a position specific manner but the extent of stabilization is smaller than the wild type Tat peptide. Apart from acetylation, lysine at position 50 and 51 is also known to undergo methylation [27] although not simultaneously thus competing for acetylation at the same residue. ^Me^P_50_ binding to HIV-1 TAR RNA was found to enhance the stability of the RNA with Δ*T*_*m*_ = 3.1 °C, (Figure 3 ^Me^P_50_) which is similar to the stabilization brought upon wild type Tat peptide binding. ^Me^P_51_ binding increases the stability of the RNA by 4 °C (Figure 3 ^Me^P_51_), which in turn is 1°C extra stabilization than wild type Tat peptide binding. Thus these results indicate an extra stabilization of the complex upon modification of the lysine residue, although the extent of stabilization varies depending on the type and position of modification.

### ITC Results

Post-translational modification of many proteins is known to modulate the specificity and the affinity of a plethora of RNA-protein complexes by interfering with the existing interactions or enhancing the critical interactions by additional new contacts for the complex formation. This change in the binding pattern can be followed in terms of thermodynamic parameters by ITC experiments. Calculations of enthalpy and entropy changes for the complex formation shows a wide range of fluctuations in their respective values pertaining to the stronger or weaker binding of modified peptides. Thermodynamic parameters corresponding to Tat-TAR RNA binding along with their binding affinity (*K*_*A*_) and stoichiometry (n) of binding are listed in the Table 1. 

**Figure 4:**
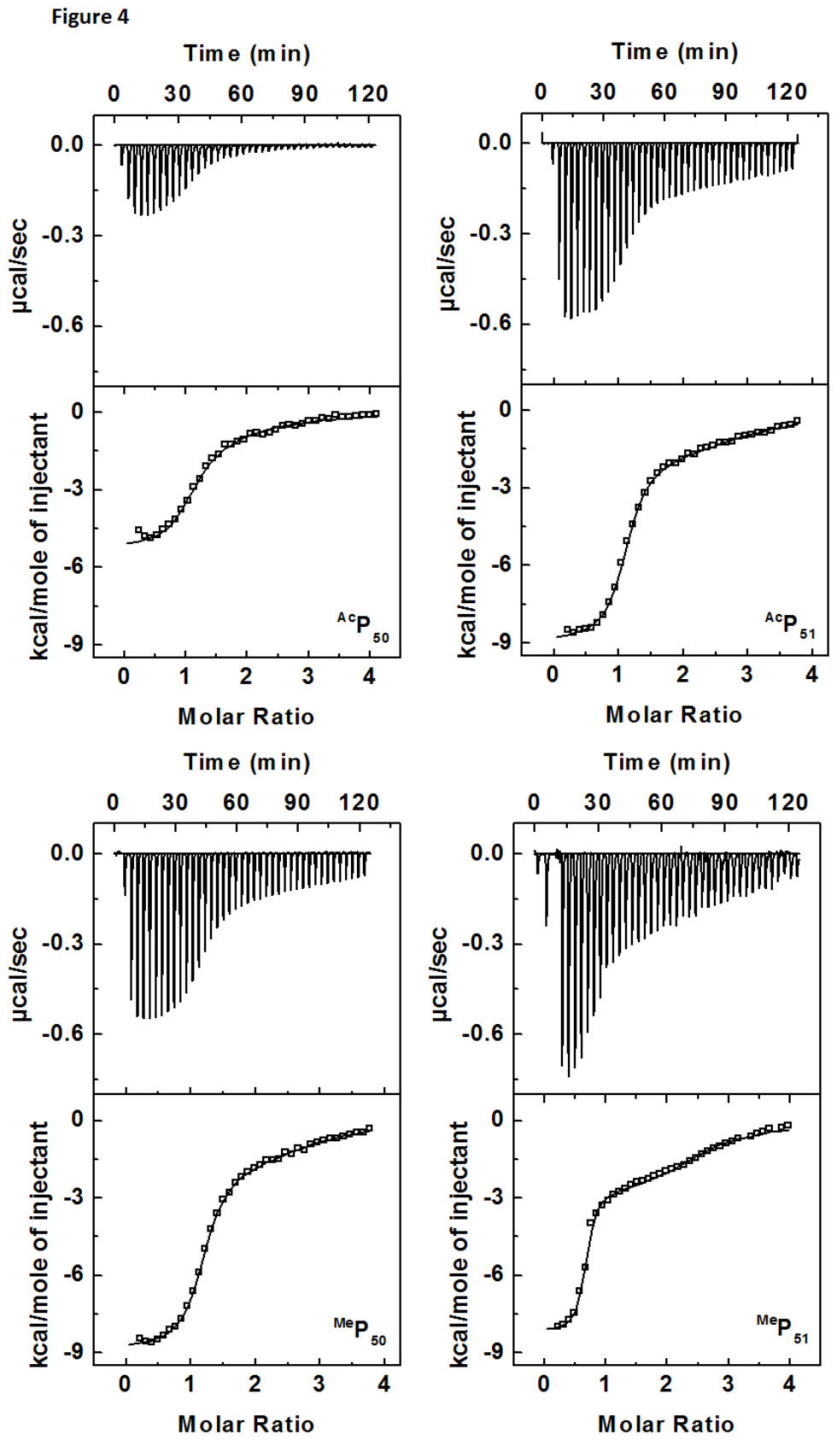
ITC titration profile of TAR RNA with Tat and different modified peptides at 25 °C. Upper panel shows the baseline corrected experimental data for peptide binding, lower panel shows the molar heats of binding (□) plotted against the peptide to RNA molar ratio. Buffer condition was as described in the caption to Figure 2. Molar heat of binding is calculated by integration of the area under the curve of each heat burst using the origin version 7.0 software (Microcal, Inc.; Northampton, MA). Fitting of ITC data (shown as solid line) was done using model for two set of binding [38] given in origin version 7.0 software (Microcal, Inc.; Northampton, MA).

Lysine acetylation at position 50 (Figure 4, ^Ac^P_50_) has a negative effect on the binding affinity of Tat peptide to TAR RNA and results in 8 fold reduction in binding affinity *K*_*A*_ = 2.3 ± 0.2 × 10^6^ M^-1^ as compared to the unmodified Tat peptide binding affinity *K*_*A*_ = 19.0 ± 0.1 × 10^6^ M^-1^ [39]. This reduction in binding affinity is also reflected in the thermodynamic components of the binding. Inspection of thermodynamic values revealed that, the enthalpic contribution of the ^Ac^P_50_ binding to TAR RNA is Δ*H* = -5.2 ± 0.2 kcal/mol almost same as wild type Tat peptide binding Δ*H* = -5.1 ± 0.1 kcal/mol. However the entropic component for ^Ac^P_50_ binding TΔS = 3.4 ± 0.2 kcal/mol was reduced significantly as compared to the unmodified Tat i.e. TΔS = 4.7 ± 0.1 kcal/mol, indicating a positive enthalpy entropy compensation which results in the increased binding affinity as compared to wild type Tat binding. Thus the change in binding affinity is originating primarily due to the reduced entropic change ΔTΔS = 1.3 kcal/mol. On the other hand ^Ac^P_51_ showed milder influence (Figure 4, ^Ac^P_51_), 3 fold reduction, on the binding affinity, *K*_*A*_ = 5.6 ± 0.4 × 10^6^ M^-1^, eventhough there is large enthalpy-entropy compensation. The enthalpy of binding for ^Ac^P_51_ is Δ*H* = -8.8 ± 0.3 kcal/mol and entropy change TΔS = 0.3 ± 0.1 kcal/mol. Contrary to acetylation, ^Me^P_50_ showed (Figure 4, ^Me^P_50_) very small decrease in the binding affinity *K*_*A*_ = 15.0 ± 0.9 × 10^6^ M^-1^ as compared to the wild type Tat peptide. Although there is a large increase in the binding enthalpy of ^Me^P_50_ Δ*H* = -8.8 ± 0.2 kcal/mol, the entropy change reduced to TΔS = 0.9 ± 0.1 kcal/mol. On the other hand ^Me^P_51_ showed 3 times increase (Figure 4, ^Me^P_51_) in binding affinity with *K*_*A*_ = 59.0 ± 0.9 × 10^6^ M^-1^ while enthalpic change of this peptide binding was more favorable with Δ*H* = -8.1 ± 0.1 kcal/mol having favorable entropic change, TΔS = 2.4 ± 0.1 kcal/mol. 

### SPR results

**Figure 5:**
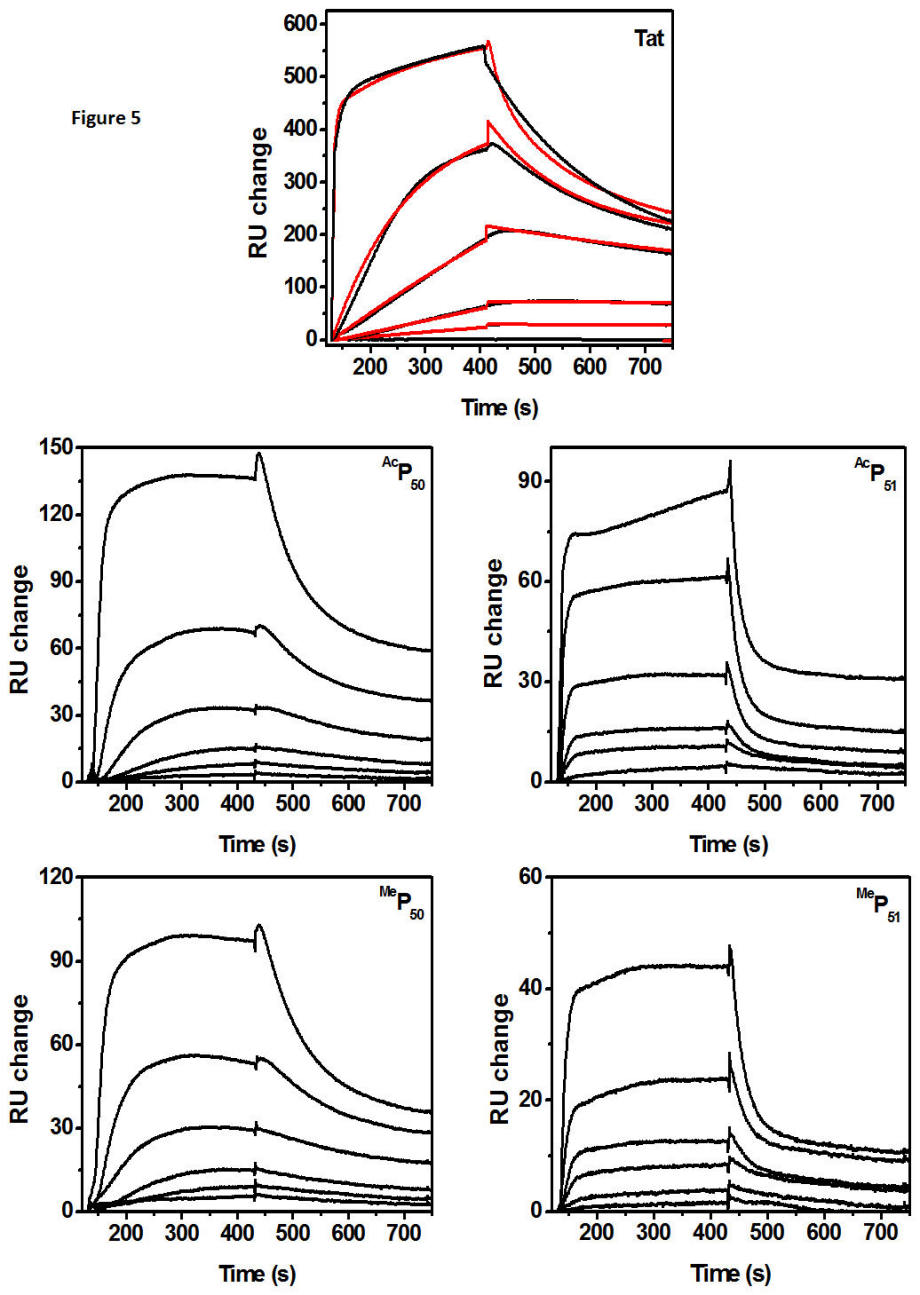
SPR sensogram showing binding kinetics of Tat peptide along with the fitting curves obtained from fittings using two independent site model. The concentration series of peptides used for studying kinetics range from 9.5 nM to 1.2 μM. The expepriments were conducted in the 10 mM sodium cacodylate buffer containing 70 mM NaCl and 0.l mM EDTA at pH 7.5. The representative fitting curves of sensogram for Tat peptide binding is shown in red color.

SPR is a powerful tool to monitor real time kinetics of binding of two partners. Here we have used it to understand the change in kinetic behavior of the binding of TAR RNA-Tat peptide interaction upon lysine modification. Analysis of the sensogram was done using model for two independent sites, and the goodness of the fittings were measured by minimizing χ^2^ value. The two binding sites observed have different binding affinities and rates of association and dissociation. The first binding site was stronger with binding affinities in the order of 10^7^ M^-1^ whereas second binding site values were of the order 10^6^ M^-1^ (Table 3). The analysis of kinetic sensogram of binding of Tat peptide (Figure 5, Tat) to immobilized TAR RNA showed association rate constant *k*_*a*_ of 2.4 × 10^4^ M^-1^s^-1^. Similarly rate constant of dissociation, *k*_*d*_ was found to be 1.9 × 10^-3^ s^-1^. The equilibrium association constant *K*_*A*_ = 1.2 × 10^7^ M^-1^, is very similar to the previously reported value obtained from other experimental techniques [39]. Different lysine modifications exhibit differential effects on various kinetic parameters. ^Ac^P_50_ showed (Figure 5, ^Ac^P_50_) a 7 fold reduction in the binding affinity *K*_*A*_ = 1.9 × 10^6^ M^-1^ with the association rate constant similar to the wild type Tat peptide binding, and more than 5 times faster rate of dissociation. The same modification at the other lysine ^Ac^P_51_, exhibited (Figure 5, ^Ac^P_51_) milder effect on the equilibrium binding affinity, *K*_*A*_ = 2.9 ± 0.4 × 10^6^ M^-1^, which was 4 times lesser than the unmodified Tat peptide. However the rate of association was 0.8 times slower, and rate constant for dissociation was nearly 4 times faster than the wild type Tat peptide. Thus acetylation at different lysine positions affect the binding affinity through different mechanisms. The equilibrium binding constants obtained from SPR experiments follow the similar trend as observed in ITC results.

**Table 3:** Effect of lysine acetylation and methylation on equilibrium and kinetic rate constants of Tatpeptide binding to HIV-1 TAR RNA.

**Peptide**	***k*_*a1*_ × 10^4^ (M^-1^s^-1^)**	***k*_*d1*_ × 10^-3^ (s^-1^)**	***K*_*A1*_ × 10^6^ (M^-1^)**	***k*_*a2*_ × 10^3^ (M^-1^s^-1^)**	***k*_*d2*_ × 10^-3^ (s^-1^)**	***K*_*A2*_ × 10^6^ (M^-1^)**
Tat	2.4	1.9	12.8	7.9	2.4	3.2
^Ac^P_50_	1.7	9.4	1.8	1.8	6.0	0.3
^Ac^P_51_	2.1	7.2	2.9	7.0	9.8	0.7
^Me^P_50_	5.6	3.5	16.0	7.9	2.4	3.2
^Me^P_51_	4.7	1.2	39.0	6.3	4.8	1.3

The error levels for the values of *k*_*a*_, *k*_*d*_, and *K*_*A*_ were within ± 10% error.

 In contrast to acetylation, lysine methylation showed almost no effect at K50 position whereas, at K51 methylation resulted in increased binding affinity. ^Me^P_50_ does not show (Figure 5, ^Me^P_50_) any significant effect on the binding affinity even though rate of association was faster, it was negatively compensated by a slower dissociation constant. In case of ^Me^P_51_ peptide binding (Figure 4, ^Me^P_51_), the binding affinity was found to increase *K*_*A*_ = 39.0 ± 0.9 × 10^7^ M^-1^ by a factor of more than 3 as compared to the unmodified Tat peptide. In this case the rate of association as well as the rate of dissociation both were increased as compared to wild type Tat peptide binding. 

## Discussion

Post translational modifications of proteins add another layer of the regulation in structure activity relationship of many RNA protein interactions. Different modifications provide additional facets to the interacting proteins which can either help in making better contacts between RNA and protein or hamper the interaction by steric clashes or negative interactions. Other than arginine methylation, Tat protein is also known to undergo lysine methylation and acetylation. Our results provide insight into the molecular understanding of how acetylation of lysine at K50 position leads to the inability of Tat peptide binding to TAR RNA [31], on the other hand methylation, another type of PTM of lysine at K51 position results in transcriptional activation [41]. Here we have shown that acetyl group when present at lysine 50 (^Ac^P_50_) stabilizes the complex by Δ*T*_*m*_ = 2.3 °C, which is significantly lower than stabilization by wild type Tat peptide binding ΔΔT_*m*_ = -0.7 °C. This lower stabilization could be a manifestation of weaker binding affinity which was found to be 8 fold lesser than the wild type Tat peptide. On the other hand ^Ac^P_51_ stabilized the TAR RNA structure by 2.7 °C with 3 fold reduction in binding affinity. These results also support the earlier findings where acetylation of Tat has been shown to reduce the binding affinity of Tat peptide to TAR RNA element and this effect is more pronounced at K50 than K51 [31]. As K50 acetylation exhibits drastic effect on the binding affinity, this could be one of the reasons why lysine at 50^th^ position is more preferred site for acetylation in the naturally occurring Tat protein. Moreover simultaneous acetylation of K50 and K51 of Tat results in copmplete abrogation of Tat-TAR RNA interaction [31]. In case of ^Ac^P_50_ the enthalpic change remains almost unaffected thus loss in binding affinity exclusively originates from entropic changes. On the other hand ^Ac^P_51_ has high enthalpy of binding ΔΔH_*1*_ = -3.7 kcal mol^-1^ (Table 2) as compared to wild type Tat peptide binding but the entropy change was very small, thus indicating enthalpy-entropy compensation for binding affinity. However the major contributor towards reduced binding affinity was reduction in entropy change. This could arise because of the entrapment of water molecules around extra acetyl group leading to reduction of favorable entropic component. Entrapment of water molecule results in reduced enthalpic weightage to RNA binding [42] but as enthalpic component remained almost unaffected, we can speculate enthalpic component compensation by extra non-covalent interaction between, water and side chain of the amino acid and RNA. The comparison of the binding parameters at the second binding site shows a reduction, irrespective of the position of modification, in the binding affinity with favorable enthalpy-entropy compensation. This clearly suggest that the second binding site is mostly electrostatic in nature and binding affinity was reduced upon modification due to loss of positive charge . Kinetic parameters also suggest ^Ac^P_50_ association rate is 0.7 times slower and the dissociation rate is 5 times faster. ^Ac^P_50_ binds to RNA with an association rate of 1.70 × 10^4^ M^-1^s^-1^ and dissociation rate constant of 9.4 × 10^-3^ s^-1^. The binding affinity was calculated as 1.8 × 10^6^ M^-1^ which is in close correlation with the binding affinity calculated from ITC experiments. Our results also suggest that the association rate of ^Ac^P_50_ is almost same as unmodified Tat peptide but the dissociation rate is 5 times faster, so the weak binding affinity of ^Ac^P_50_ peptide could directly be ascribed to the faster dissociation rate. Although lysine acetylation was found to decrease the binding of the Tat peptide in position specific manner, methylation of lysine showed opposite effects. We found methylationof K50 (^Me^P_50_), did not show any extra stabilization ΔΔT_*m*_ = 0.1 °C whereas for K51 (^Me^P_51_) binding ΔΔT_*m*_ = 1.0 °C. This could be the result of position specific effect of the lysine methylation. These results also suggested similar binding of ^Me^P_50_ peptide to TAR RNA, as wild type Tat peptide. This was also supported by binding affinity values of ^Me^P_50_ peptide calculated from ITC experiments which was similar to wild type Tat peptide binding to TAR RNA. Although there is a significant amount of increase in favorable binding enthalpy of the ^Me^P_50_, the decrease in favorable entropy negatively compensates it, leaving the binding affinity unaffected. On the other hand, the reduction in the favorable entropic component of the binding can be explained in terms of change in hydration as in case of ^Ac^P_50_. Similar to ^Me^P_50_ binding, ^Me^P_51_ also showed enthalpy entropy compensation but the favorable entropic component dominates, resulting in higher binding affinity of ^Me^P_51_. Large change in thermodynamic binding parameters in case of ^Me^P_50_ implicates that binding leads to a large amount of molecular rearrangement in peptide and RNA, retaining the binding affinity similar to unmodified Tat peptide. The binding affinity at second binding site was reduced to different extent at two positions *KA2*/ *K*_*A2*_^Tat^ = 0.24 for ^Me^P_50_ and 0.68 for ^Me^P_51_ again supporting the electrostatic nature of the second binding site. On the second binding site also there is a large enthapy-entropy compensation for the reduced binding affinty. Kinetic parameters suggest a 2 times faster association rate for both ^Me^P_50_ and ^Me^P_51_ peptides, but the dissociation rate was 2 times faster in case of ^Me^P_50_ peptide, which cancels the favorable effect of faster rate of association leading to the binding affinity similar to un modified Tat peptide. However in case of ^Me^P_51_ peptide the rate of dissociation is similar to the unmodified Tat peptide so the faster association rate contributes favorably to the binding affinity. Thus these results altogether suggest that methylation when present at lysine 51 increases the binding efficiency of the peptide by modulating the kinetic as well as thermodynamic component of the binding. Our results also provide the thermodynmic and kinetic explanation for the enhancement of transactivation by monomethylation at K51 by cellular lysine methyltransferases Set7/9-KMT7 [41]. It shows that monomethylation at K51 results in stronger binding of Tat protein to TAR RNA element leading to transcriptional activation. Similar results had been observed in case of N,N-dimethylation of lysine residue of Rev peptide which increases the binding along with the improvement of specificity to Rev response element (RRE) of HIV-1 in a position dependent manner [43]. 

## Conclusion

Here we have shown the position specific effect of N-acetylation and N-methylation of lysine on the binding of Tat peptide to HIV-1 TAR RNA. Our results indicated that acetylation of lysine results in reduction of binding affinity of the peptide to RNA in a position specific manner. The molecular changes occurring in response to lysine acetylation induced almost similar structural perturbation in RNA as wild type Tat peptide. The decrease in the binding efficiency could be attributed to the reduction in favorable entropic component for acetylated K50. However in case of K51 acetylation, although there is a large increase in favorable enthalpy of binding it is negatively compensated by the decrease in favorable entropy, thus leading to milder effect on the binding affinity. Similarly in case of N-methylation of lysine binding affinity remain unaffected at K50 but increases at K51, though there is a large enthalpy-entropy compensation happening in both the cases. Further our results also demonstrate the tradeoff between the rates of association and dissociation to give diverse outcomes in response to different modifications in a position specific manner. Thus in conclusion our results provide valuable insights into the molecular understanding of the effect of post translational modification of lysine in RNA binding proteins.
